# Single domain antibody: Development and application in biotechnology and biopharma

**DOI:** 10.1111/imr.13381

**Published:** 2024-08-21

**Authors:** Ting Yu, Fang Zheng, Wenbo He, Serge Muyldermans, Yurong Wen

**Affiliations:** ^1^ Center for Microbiome Research of Med‐X Institute, Shaanxi Provincial Key Laboratory of Sepsis in Critical Care Medicine, The First Affiliated Hospital Xi'an Jiaotong University Xi'an China; ^2^ The Key Laboratory of Environment and Genes Related to Disease of Ministry of Education, Health Science Center Xi'an Jiaotong University Xi'an China; ^3^ Laboratory of Cellular and Molecular Immunology Vrije Universiteit Brussel Brussels Belgium

**Keywords:** biotechnological tools, diagnosis, heavy chain antibodies, nanobody, single‐domain antibody, therapeutics, VHH

## Abstract

Heavy‐chain antibodies (HCAbs) are a unique type of antibodies devoid of light chains, and comprised of two heavy chains‐only that recognize their cognate antigen by virtue of a single variable domain also referred to as VHH, single domain antibody (sdAb), or nanobody (Nb). These functional HCAbs, serendipitous discovered about three decades ago, are exclusively found in camelids, comprising dromedaries, camels, llamas, and vicugnas. Nanobodies have become an essential tool in biomedical research and medicine, both in diagnostics and therapeutics due to their beneficial properties: small size, high stability, strong antigen‐binding affinity, low immunogenicity, low production cost, and straightforward engineering into more potent affinity reagents. The occurrence of HCAbs in camelids remains intriguing. It is believed to be an evolutionary adaptation, equipping camelids with a robust adaptive immune defense suitable to respond to the pressure from a pathogenic invasion necessitating a more profound antigen recognition and neutralization. This evolutionary innovation led to a simplified HCAb structure, possibly supported by genetic mutations and drift, allowing adaptive mutation and diversification in the heavy chain variable gene and constant gene regions. Beyond understanding their origins, the application of nanobodies has significantly advanced over the past 30 years. Alongside expanding laboratory research, there has been a rapid increase in patent application for nanobodies. The introduction of commercial nanobody drugs such as Cablivi, Nanozora, Envafolimab, and Carvykti has boosted confidence among in their potential. This review explores the evolutionary history of HCAbs, their ontogeny, and applications in biotechnology and pharmaceuticals, focusing on approved and ongoing medical research pipelines.

## 1 INTRODUCTION

Immunity is a crucial development helping animals to defend against external threats. While innate immunity is present across all species, adaptive immunity was traditionally thought to be exclusive to vertebrates. However, recent discoveries have found evidence of adaptive immunity in invertebrates, like flies, suggesting that this adaptive immunity may exist in various forms across different species.^1^

About 30 years ago, the group of Prof. R. Hamers made the serendipitous discovery in camelids of Heavy chain‐only antibodies (HCAbs) devoid of light (L) chains.^2^, ^3^ These unique antibodies are a type of IgG isotype (IgG2/IgG3), and they can even reach 81% of the total IgG content in sera of the *Camelus* genus.^4^ The IgG is the most abundant immunoglobulin in human humoral immunity,^5^ indicating that HCAbs are vital for the immune protection of camelids. While HCAbs also occur naturally in species like cartilaginous fish, mice, and even humans, only camelids and cartilaginous fish have functional HCAbs that effectively bind to antigens. In mice and humans, these antibodies may sometimes lead to disease or immune system disorders.^6^, ^7^, ^8^

The emergence of HCAbs in camelids came as an unexpected surprise, as it challenged the traditional view of the immunoglobulin hetero‐tetrameric structure, invariably composed of paired heavy (H) and light (L) chains.^9^ This finding offered intriguing opportunities to explore how immune systems might evolve under evolutionary pressures.

Scientists made major efforts investigating this intriguing evolutionary event, but a definitive answer remained elusive. However, it became clear that it was an independent evolutionary event, occurring after the appearance of conventional hetero‐tetrameric antibodies. The major driving force promoting this adaptive immunoglobulin evolution must have been an infection by a devastating viral disease, possibly enhanced by factors such as extreme climate conditions such as extreme temperatures and water scarcity. The heavy chain‐only antibodies found in cartilaginous fish, known as immunoglobulin new antigen receptor (IgNAR) definitely emerged much earlier than the camelid HCAbs. Nevertheless, the camelid HCAbs underwent a striking molecular convergent evolution with IgNAR.^10^ Exploring the potential evolutionary link between these two species may provide insights into the development of camelid antibodies.

The variable antigen‐binding domains of HCAbs, known as nanobodies, have seen a continuously expanding range of applications over the years. As novel class of therapeutic agents, nanobodies of about one‐tenth of the size of conventional antibodies, show versatility in biomedical applications. In cancer treatment, they can be tailored to target tumor‐specific markers, aiding in finding and locating the tumor sites, or in enhancing drug delivery and minimizing off‐target effects. Their ability to cross the blood–brain barrier is particularly advantageous for treating neurological disorders. Beyond oncology and neurology, nanobodies are also applied in infectious diseases and diagnostics, highlighting their multifunctionality across various medical fields.

Since their discovery, nanobodies, have transitioned from laboratory research to practical therapeutics. The overwhelming patent applications in the United States and Europe, along with the market introduction of the first nanobody drug, Cablivi, in 2019, has undoubtedly significantly advanced this field. This review will discuss the latest developments in nanobody research and applications.

## 2 WHAT CAUSES THE EMERGENCE OF HCABS IN CAMELIDS? INVESTIGATING THE UNDERLYING CAUSES

The evolution of genes is driven by mechanisms such as random mutations, gene flow, genetic drift, and natural selection. These mechanisms collectively shape the genetic nature of populations over time, probably enabling camelids to respond and adapt to changing pathogenic drifts and environments.

### 2.1 Natural selection

The appearance of jawed vertebrates marked a significant evolutionary milestone. These animals from cartilaginous fish up to mammals employ immunoglobulins (Igs) to install an antigen‐recognition capacity.^11^, ^12^ Cartilaginous fish, which appeared around 450 million years ago, are the oldest species known to produce Igs. In contrast, the first camelids, protylopus, emerged approximately 45 million years ago in North America, evolving in Tylopoda from the Artiodactyla order, which further includes Suiformes (pigs, hyppopotamidae) and Ruminantia (giraffes, deer, antilope, cattle, sheep…). However, functional HCAbs have only been detected in camelids (including the *Camelus*, *Lama*, and *Vicugna* genera), encompassing seven extant species (Arabian camel, Bactrian camel, Wild Bactrian camel, llama, guanaco, alpaca, and vicugna).^13^

The theory of natural selection suggests that camelids may have developed their unique immune system based on a dichotomic antibody structure (heterotetrameric conventional antibodies and homodimeric HCAbs) in response to viral threats possibly aggravated by harsh environmental conditions.

Viral infestations most likely have provoked the emergence of HCAbs derived from conventional H chain genes in camelids. Viruses exert selective pressures on hosts, and HCAbs are effective in neutralizing viruses and toxins, which are sometimes difficult targets for tetrameric antibodies.^14^, ^15^, ^16^ Old world camels are known to be infected by various viruses, including camelpox, influenza, and diarrhea viruses, and they carry fatal viruses like SARS and MERS.^17^, ^18^, ^19^ The VHH domain of HCAbs has shown great neutralizing potential against these viruses, highlighting its role in combating significant threats to animal health.^20^, ^21^

However, additional drivers might have assisted the emergence of the camelid HCAbs. Heat stress, which compromises immunity, is common in wildlife and livestock when ambient temperatures exceed 27°C.^22^ Camelids, however, are heat‐tolerant and can endure temperatures over 40°C.^23^, ^24^ Camelid lymphocytes have shown higher resistance to heat stress in vitro, and HCAbs from IgG2 and IgG3 subclasses exhibit greater tolerance to extreme heat than tetrameric IgG1.^14^ Some autonomous camelid HCAb variable domain (VHH) have been reported to withstand temperatures up to 90°C and those with a mean melting temperature (Tm) of 67°C regained antigen‐binding after thermal denaturation and reversible refolding.^25^

Furthermore, camelid species from more extreme environments, such as the deserts and steppes of Central Asia and North and East Africa, produce a higher quantity of HCAbs compared to those from less extreme environments, such as the high Andean regions.^26^ The proportion of VHH gene expression in wild Bactrian camels is higher than in domesticated individuals, indicating an adaptive gene expression under natural conditions.^25^ The elimination of light chains in camelid antibodies also reduces the risk of light‐chain deposition disease, which can cause kidney dysfunction, thus ensuring better water retention and reducing immune disorders under extreme temperatures.^27^, ^28^

### 2.2 Adaptive complementary antigen‐recognition strategies

Investigating the genetic and structural key elements of HCAbs might provide additional insight into its evolutionary adaptive strategies. Like most mammals, camelids utilize V(D)J recombination to generate antibody diversity. These elements are encoded by the *IGHVH(VHH)‐IGHD‐IGHJ* gene cluster. Among these, *IGHD* and *IGHJ* sets are shared to generate HCAb and heterotetrameric antibodies (H_2_L_2_), whereas *IGHVH* genes are unique in all camelids and are playing a crucial role in the formation of the antigen‐binding region of HCAbs. Both *IGHVH* and *IGHV* belong to the *IGHV3* family. All these elements are clustered in one locus on the genome.^29^, ^30^ Nucleotide diversity analysis of the *IGHVH* gene across all existing camelid species suggests a common ancestor, indicating that *IGHVH* likely evolved before the divergence of ancient camel species into the distinct lineages recognized today. Sequence analysis among alpacas, Bactrian camels, and dromedaries reveals that alpaca's *IGHVH* gene diverged somewhat from that of the *Camelus* species, likely due to a genetic flow event.

HCAbs have also evolved through mutation and genetic drift, allowing them to assemble according to a simpler homomerization and to perform as effectively, if not better, than hetero‐tetrameric antibodies. The variable region (VHH) of HCAbs shows significant mutation and drift, with framework regions (FR) contributing to the loss of the VL domain through hydrophilic mutations substituting the conserved hydrophobic amino acids in the FR2 used to interact with the variable domain of the L chain (VL).^31^ In addition, all the IGVH genes in Bactrian camel encode a cysteine in their CDR1 loop (or at position 50 according to IMGT numbering), which leads to a non‐canonical disulfide bond formation with a somatically generated cysteine in the CDR3 loop. The complementarity‐determining regions (CDR) of VHHs also exhibit a wider variety of loop conformations. Also, the CDR3 of VHHs, which is in camels on average longer than in human or mouse VHs, will undoubtedly increase antigen affinity despite the absence of the antigen binding loops provided by VLs. Additionally, increased mutation hotspots in *IGHVH* leading to increased frequency of somatic hypermutation might further diversify the antigen binding repertoire, while longer hinge regions may also enhance epitope access by HCAbs.^32^ The mutation from a conserved Leu, interacting intimately with the CH1 domain in conventional H chains, to hydrophilic Ser at position 12 of the camel *IGHVH* (but not the llama or alpaca IGVH) is expected to enhance the solubility of the autonomous VHH.^33^, ^34^, ^35^

**FIGURE 1:**
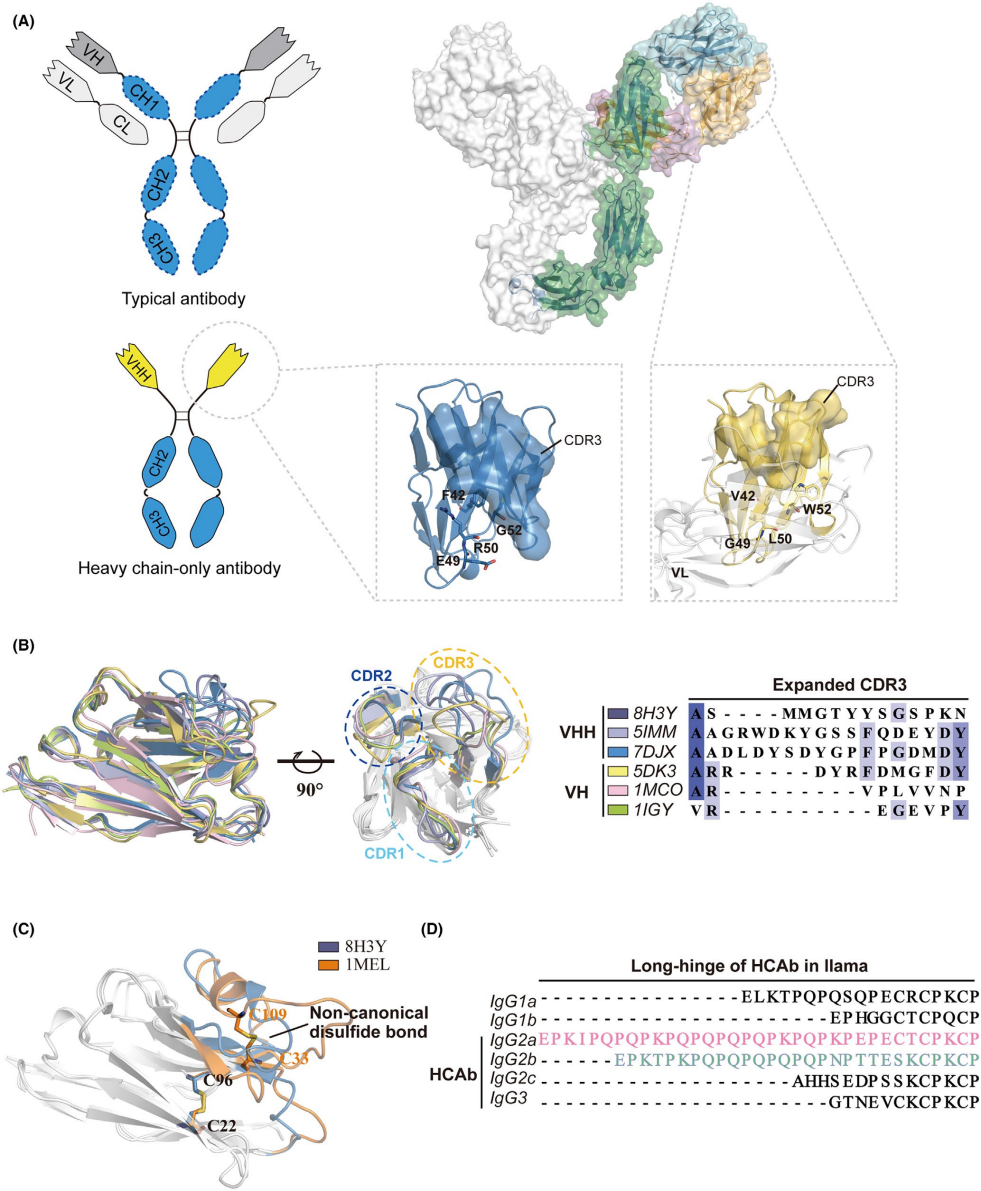
Adaptive evolution of HCAbs and nanobody structures. (A) Schematic representation of typical antibody and HCAb (left); Structural organization of intact IgG, VH (PDB: 5DK3), and VHH (PDB: 7DJX) (right). Characteristic amino acid substitutions are shown in cartoon pattern and the CDR3 region is shown in surface view (bottom right). (B) Structural alignment of VHH region from Camelid (Modena, PDB:8H3Y; Lilac, PDB:5IMM; Blue, PDB:7DJX) and VH region from Homo sapiens (Yellow, PDB:5DK3; Pink, PDB:1MCO) and Mus musculus (Green, PDB:1IGY). The right showed the sequence alignment of CDR3 regions. (C) Structural alignment of two different nanobodies, containing an additional non‐canonical disulfide bond. (Orange, PDB:1MEL). (D) Amino acid sequences of long hinge regions from rearranged llama IgG transcript (Modified from Kevin et.al^47^).

The loss of the CH1 domain in camelid HCAbs is attributed to a point mutation (G > A) at the 5′ end of the intron between CH1 and hinge exons. This mutation is conserved in all camelid *IGHG* genes used to produce HCAbs. The absence of a CH1 domain in the H chain prevents the BiP binding chaperone proteins to interact with the H chain and to retain this H chain in the Golgi before being replaced by the L chain enabling their secretion from cells.^36^, ^37^, ^38^ This adaptation, alongside the hydrophilic transformation of amino acids in the FR2 domain, supports HCAb solubility^39^, ^40^, ^41^, ^42^ **(**Figure 1A **)**. The expanded CDR loops in VHHs, particularly the extended CDR1 and CDR3 regions, compensate for the lack of VH‐VL combinatorial diversity.^15^ The longer CDR3 regions, averaging 17–18 amino acids in Bactrian camels and llamas, adapt structurally to enhance antigen affinity, including to those with cryptic epitopes **(**Figure 1B **)**.^32^, ^43^, ^44^, ^45^, ^46^, ^47^, ^48^ Non‐canonical cysteines in VHHs form additional disulfide bonds, stabilizing the long CDR3 loop and increasing antigen affinity **(**Figure 1C **)**.^49^, ^50^ The extended CDR loops and additional cysteine bridges are also observed in other species like the platypus and shark.^51^ Additionally, the hinge regions in camelid HCAbs, which vary from long and flexible to short and hingeless, significantly influence antigen binding by reducing steric hindrance**(**Figure 1D **)**.^47^ Somatic hypermutation (SHM) further enhances antigen recognition by increasing mutation rates in the variable regions of HCAbs, promoting the emergence of novel paratopes and maintaining antibody diversity despite the absence of light chain variable domains.^52^

### 2.3 Insights from Cartilaginous Fish Species in Shaping Adaptive Immune Systems

A convergent evolution explains the independent development in different species of similar traits such as a toxin resistance. HCAbs emerged first in cartilaginous fish, including sharks' IgNAR and holocephalin IgM HCAbs. HCAbs with similar sequence imprints and structural characteristics also appeared much later in camelids.^53^ Sharks, the earliest vertebrates with an adaptive immune system, developed unique IgNARs with a single variable domain (VNAR) responsible for antigen binding.^54^ The secretory IgNAR includes multiple constant domains mediating effector functions, while the transmembrane subtype lacks CH2 and CH3 domains. The VNAR domain features hypervariable loops connected via covalent disulfide bonds.^55^, ^56^ Shark VNARs exhibit adaptive structural changes, in the conserved regions including polar and charged amino acids at solvent‐exposed regions, improving solubility and the VHH of camelid HCAbs seem to have mimicked this feature.^57^ Despite different gene loci for encoding these antibodies, the convergent evolution of HCAbs in sharks and camels suggests significant evolutionary advantages in their habitats, such as high salinity and varying pH levels for sharks. Additionally, nanobodies in both species can be maternally transmitted, aiding neonatal immune development. For instance, IgG HCAb isotypes are found in alpaca colostrum, and both IgM and IgNAR are present in nurse shark eggs, highlighting their crucial role in the immune system.^58^, ^59^

## 3 APPLICATIONS

### 3.1 Expansion and commercialization of nanobodies

In just three decades since the discovery of camelids HCAbs, their variable antigen‐binding regions, known as VHHs or nanobodies, have found applications in multiple fields. Their small size, stability, and high antigen affinity have encouraged significant commercial interest, with patent applications increasing rapidly worldwide, particularly in the past decade.^60^, ^61^, ^62^

**FIGURE 2:**
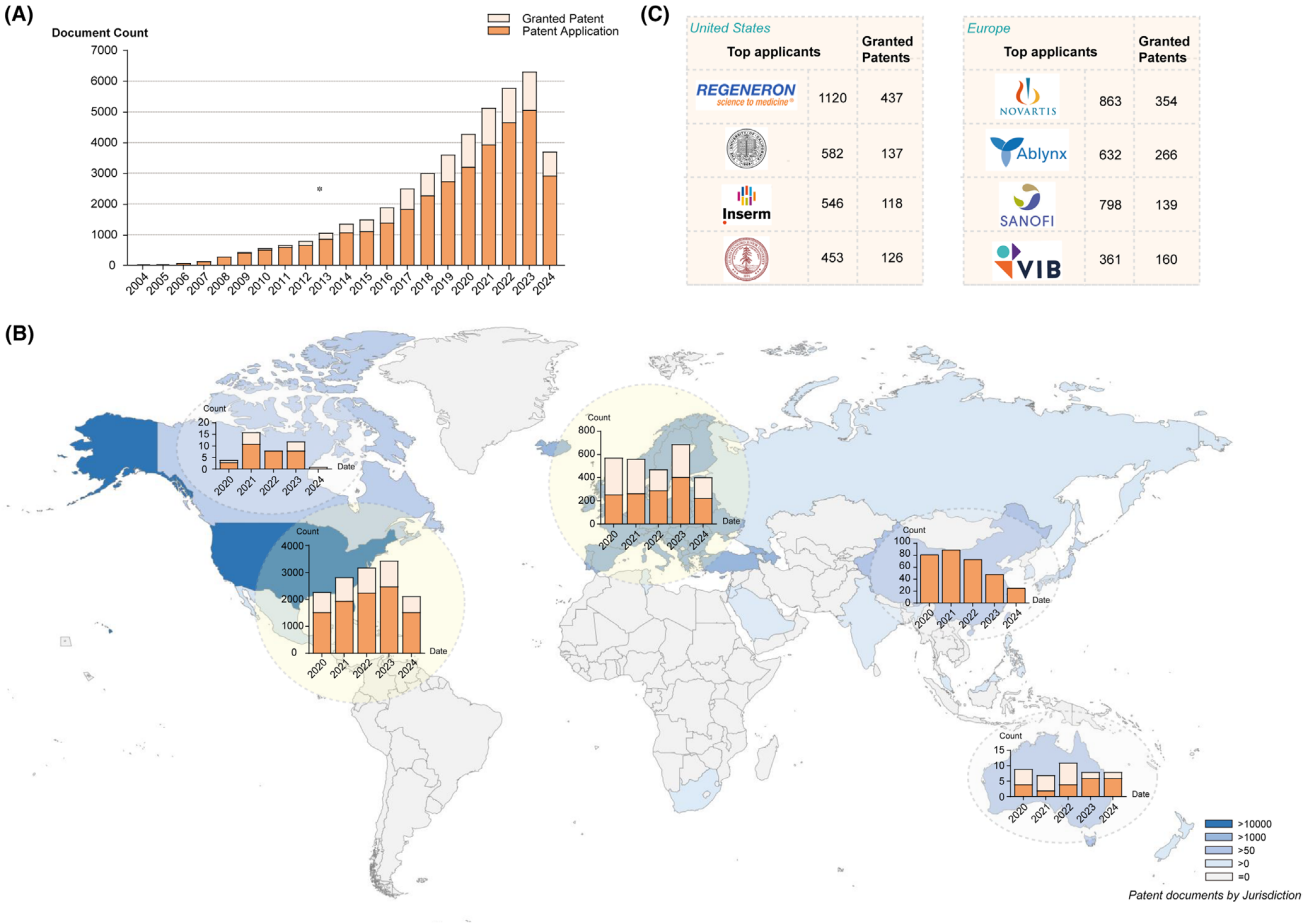
Search results of the LENS.ORG patent database by applying the terms “nanobody” (https://www.lens.org/, accessed on 19 July 2024). (A) Annual patent trends related to nanobody. (B) Patent documents by Jurisdiction and trend over 5 years. (C) Top applicants from North America and Europe.

In 2014, global nanobody patent applications exceeded 1000 for the first time, with a 20% yearly increase since then **(**Figure 2A **)**. This remarkable increase was possibly partly driven by the positive Phase I results of Ablynx's lead therapeutic nanobody (Caplacizumab), the first proof‐of‐concept for nanobody application in medicine. The expiration of the first product claims on HCAb fragments from the ‘Hamers’ patents in Europe (2014) and the United States (2017) further accelerated the emergence of camelid single‐domain antibodies (sdAbs) as mainstream biotherapeutics.^62^ The number of granted patents surpassing 1000 by 2020, witnesses that the nanobody industry is entering a phase of robust growth **(**Figure 2A **)**.^63^

Currently, the majority of nanobody patent applications are concentrated in the United States with an average of over 1000 patent applications per year, followed by Europe and China **(**Figure 2B **)**.^63^ Canada and Australia also rank highly, although their annual number of patent applications remain below 20 **(**Figure 2B **)**.^63^ The regional dominance of nanobody patents underscores their pivotal roles in advancing research and commercialization.

The prominent status of the United States and Europe in the number of nanobody patent applications is also reflected in the numerous patents held by biopharmaceutical companies based in these regions **(**Figure 2C **)**.^63^ Biopharmaceutical companies like Regeneron Pharmaceuticals in the United States, with over 1000 patent applications and more than 400 granted patents and Novartis AG and Ablynx NV in Europe each holding over 250 granted patents **(**Figure 2C **)** ^63^ demonstrate the leading position of these companies in the nanobody development. Furthermore, the rising number of patents reflects increased interest and substantial investments in nanobody research and development. Collaborations between academia, biotech spin‐offs, and pharmaceutical companies are expected to further accelerate advancements in this field.

### 3.2 Advancements in nanobody Production and Applications

**FIGURE 3:**
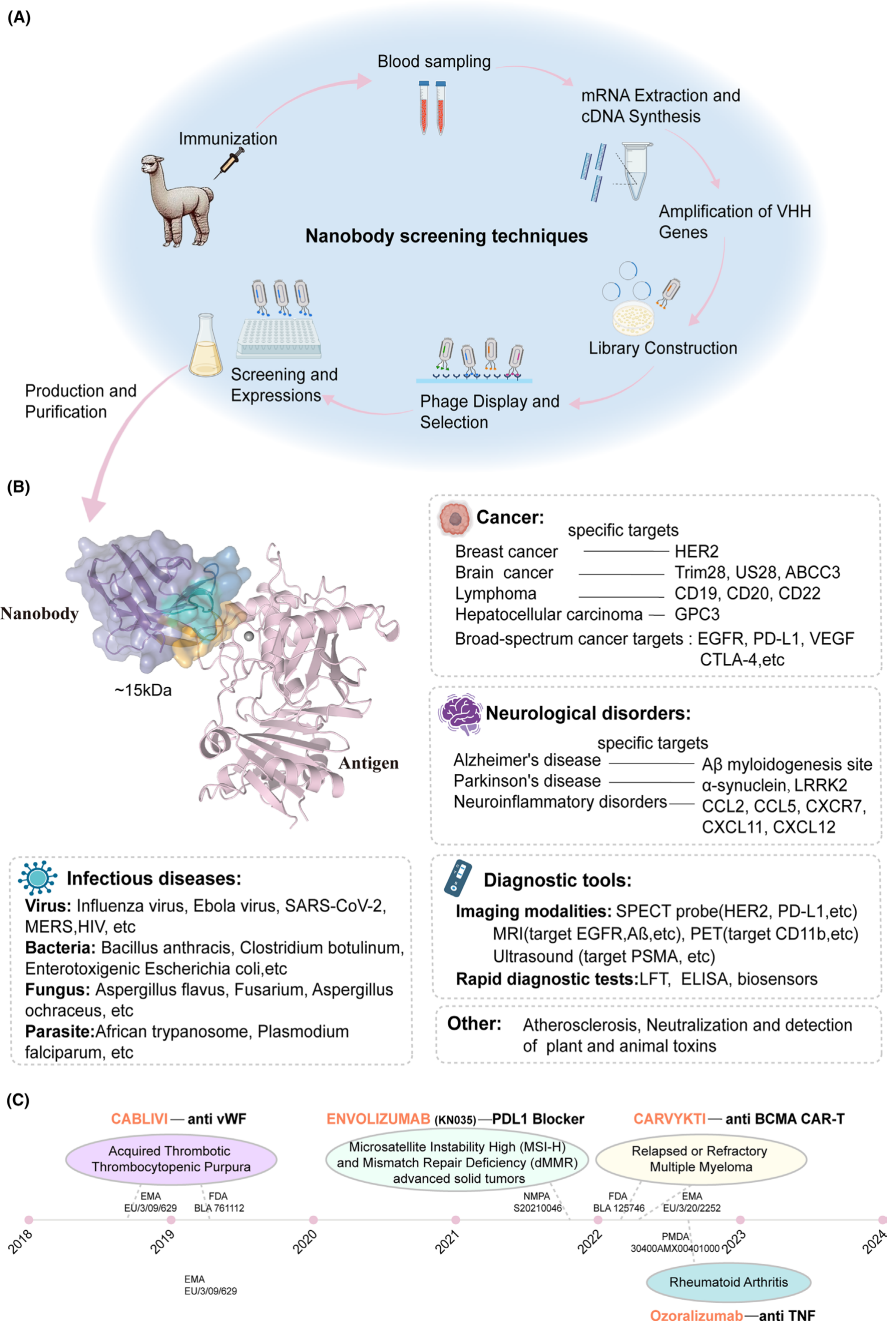
(A) Schematic illustration of regular nanobody production operations using a phage display library. It involves immunizing an alpaca with the target antigen, followed by blood sampling to isolate peripheral blood lymphocytes (PBLs). mRNA is extracted from PBLs and reverse‐transcribed into cDNA. VHH genes are amplified via PCR and inserted into a phage display vector to create a library. Phages displaying unique VHHs undergo selection for antigen binding through panning. Selected phage clones are screened, and top candidates are subcloned into expression vectors. The VHHs are produced in host cells like *E. coli* and purified using affinity chromatography. Created with BioRender.com (B) Structural diagram of nanobody binding to the antigen (PDB: 8H3Y) and summary of the important applications and related targets of Nbs in the realm of medicine, especially on cancer, neurological disorders, infectious diseases and diagnostic tools. Created with BioRender.com (C) Timeline of approved therapeutic nanobodies. The names of the products are colored orange, followed by its target. The marketing authorization number is on the dotted line.

The identification and production of nanobodies are well‐established as emphasized by the steadily growing number of academia, research institutes, and biotech companies using these technologies. The construction of nanobody libraries and their screening techniques to retrieve potent monoclonal nanobodies are very efficient involving bacterial, yeast, and mammalian cell expression systems,^64^, ^65^, ^66^ which were already reviewed previously.^67^ These techniques typically starts with the immunization of a camelid (e.g., llama, alpaca, or dromedary) or transgenic mice (equipped with VHH germline genes and adapted constant genes lacking the first constant domain) with the antigen of interest to elicit a HCAb immune response and solating lymphocytes from the immunized animal's blood. Next, RNA is extracted from these lymphocytes and converted into cDNA. This cDNA serves as a template to amplify the nanobody genes by PCR. The amplified gene fragments are ligated into a dedicated selection vector for phage display or yeast display. Transformation into the appropriate host cells, display on phage or yeast are used to retrieve the antigen‐specific nanobodies. These retrieved nanobodies are then introduced into an appropriate host, often *E.coli*, for nanobody expression. The expressed nanobodies are harvested from the bacterial culture and purified, typically through affinity chromatography using the antigen or specific purification tags preferably fused at the C‐terminal end of the nanobody. Finally, the purified nanobodies undergo characterization and validation tests to confirm their specificity and binding characteristics to their cognate target antigen **(**Figure 3A **).** While immune nanobody libraries are the standard for most selections, synthetic libraries or naïve nanobodies are becoming increasingly popular. Also, alternative selection methods based on deep sequencing or mass‐spectrometry identification have been designed.

Purified nanobodies have widely diverse applications, including cancer treatment and brain‐related diseases^68^, ^69^; bioscience, where they are used in sophisticated imaging of, or modulating presence and function of intracellular antigens^57^, ^70^, ^71^; and even in plant science, enhancing resistance to plant pathogens.^72^ However, the most significant impact of nanobodies has been in the realm of medical diseases. As a novel class of therapeutic antibodies that are much smaller and more stable than conventional antibodies, nanobodies have the unique capacity to extravasate from veins and diffuse readily into tissue to reach tumor‐specific antigens. For examples, in the case of breast cancer,^73^ studies are mostly targeting the human epidermal growth factor receptor 2 (HER2) tumor biomarker often overexpressed on the surface of breast cancer cells.^74^, ^75^ As for hepatocellular carcinoma (HCC),^76^ nanobodies that target the Glypican‐3 (GPC3) signaling pathways have assisted to regulate the proliferation and metastasis of HCC cells.^77^, ^78^ When it comes to brain tumor research, studies are focusing on glioblastoma, targeting therapeutic markers such as transcription factor Trim28,^79^ chemokine receptor US28,^80^ and ATP Binding Cassette subfamily C member 3 (ABCC3).^81^ Additionally, nanobodies are applied to target lymphoma, including but not limited to CD19, CD20, and CD22.^82^, ^83^, ^84^ Some research also involves the broad‐spectrum targets, such as the epidermal growth factor receptor (EGFR),^61^, ^85^, ^86^ programmed cell death ligand‐1 (PD‐L1),^87^, ^88^ vascular endothelial growth factor (VEGF),^89^, ^90^ and cytotoxic T lymphocyte‐associated protein‐4 (CTLA‐4),^91^, ^92^ which are critical in the development and progression of various cancers. Conversely, the reports on the unique ability of nanobodies to permeate the blood–brain barrier offers a significant advantage in the treatment of neurological disorders.^93^ This is particularly pertinent in the delivery of therapeutics for diseases such as Alzheimer's,^94^ where they can be directed to the Aβ amyloidogenic sites.^95^ Similarly, in Parkinson's disease, nanobodies can be employed to specifically target α‐synuclein^96^, ^97^ and leucine‐rich repeat kinase 2 (LRRK2),^98^, ^99^ which are specific pathogenic targets of the disease. Moreover, nanobodies also hold promise in combatting neuroinflammatory disorders by specifically targeting chemoattractant cytokine ligands (CCL2, CCL5, CXCR7, CXCL11, and CXCL12), which aims to modulate the immune system components and tackle the inflammation by inhibiting pro‐inflammatory cytokines or their receptors.^93^, ^100^ (Figure 3B).

Beyond the applications in oncology and neurology, nanobodies have demonstrated in the past decade remarkable versatility and expanded their utility to the fields of infectious diseases, detection and neutralization of biotoxins, and disease diagnosis.^101^, ^102^, ^103^ In infectious diseases, nanobodies can neutralize pathogenic microorganisms by binding to key proteins involved in the pathogen's entry into host cells. This has been particularly highlighted in the case of viruses.^104^ For example, in the case of influenza virus, researchers have engineered nanobodies that can attach to the hemagglutinin protein on the virus's surface, a pivotal protein for the virus' entry into the cell.^105^, ^106^ Similarly, during the COVID‐19 pandemic, scientists developed nanobodies that targeted the SARS‐CoV‐2 virus' spike protein,^107^, ^108^, ^109^ which is crucial for viruses to enable the entrance into human cells via the ACE2 receptor. For HIV, nanobodies have been developed to associate with the CD4 binding site of the virus' envelope protein, gp120 and gp41, inhibiting its ability to infect T‐cells.^110^, ^111^ Moreover, nanobodies have been explored as potential drugs to reduce viral hemorrhagic fevers, such as Ebola,^112^ by targeting and neutralizing the glycoproteins involved in virus entry into cells. In addition, nanobodies have found numerous applications in the detection and neutralization of biotoxins of bacteria, like anthrax toxin from *Bacillus anthracis*,^113^, ^114^ botulinum toxin produced by *Clostridium botulinum* ^115^ and toxins produced by *Enterotoxigenic Escherichia coli* (ETEC).^116^, ^117^, ^118^ They are also being applied for combating mycotoxins, including aflatoxin, fusarium toxin, and ochratoxin.^119^ For parasitic diseases, one anti‐VSG nanobody has been found inducing membrane fission and remodeling in African trypanosomes,^120^ and another Pfs230‐specific nanobody showed significant blocking of the transmission of *P. falciparum* and reduction in the formation of flagellation centers.^121^ (Figure 3B).

### 3.3 Diagnostic applications of nanobodies in medical imaging and rapid testing

nanobodies are increasingly used in diagnostics due to good biodistribution and their high affinity for disease biomarkers. As such, nanobodies have been coupled to a range of modalities for in vivo imaging to obtain sharp visualization of tumors^122^ by MRI probes for detecting Alzheimer's disease^123^ or radiotracers in combined positron emission tomography (PET) and magnetic resonance imaging (MRI) in the study of atherosclerosis.^124^, ^125^ Near‐infrared nanobody probes and Fluorescent nanobodies that unveil the oncogenic loci of colon or pancreatic cancers have also been reported.^126^, ^127^, ^128^ These applications enhance the detailed images to better understand and monitor the disease and enable earlier and more accurate diagnosis.

**Single‐photon emission computed tomography (SPECT)** utilizes gamma rays emitted by radioactive isotopes such as ^99m^Tc and ^111^In. This imaging modality is applied to visualize various targets, including amyloid deposits, immune cell types, and specific disease markers. For example, ^99m^Tc‐labeled anti‐HER2 nanobodies have been used to image HER2‐positive tumors,^129^ and ^99m^Tc‐labeled anti‐VCAM1 nanobodies have been employed to detect atherosclerotic plaques.^130^ SPECT offers high sensitivity and specificity, making it suitable for deep tissue imaging. However, it is limited by its moderate spatial resolution, which ranges from 8 to 10 mm.

**Positron emission tomography (PET)** employs positron‐emitting radioisotopes such as ^18^F, ^68^Ga, and ^89^Zr. These nuclides when attached to nanobodies are particularly useful for imaging cancer biomarkers like HER2,^131^ HER3,^132^ and EGFR,^133^ as well as immune checkpoints such as PD‐L1^134^ and CTLA‐4,^135^ and the tumor microenvironment. PET offers higher spatial resolution and sensitivity compared to SPECT, enabling real‐time tracking of biological processes. However, the technique requires a cyclotron for isotope production, which contributes to higher costs.

**Optical imaging (OI)** utilizes fluorescent dyes for visualization, making it suitable for imaging surface lesions, intraoperative imaging, and endoscopic examinations. This non‐radioactive technique is flexible and relatively inexpensive. For example, anti‐HER2 nanobodies conjugated with IR‐Dye 800CW have been used for imaging HER2‐positive tumors,^136^ and anti‐EGFR nanobodies labeled with IR‐Dye700DX have been employed to visualize EGFR‐expressing cells.^137^ These nanobody‐based probes enhance the specificity and sensitivity of OI, allowing for precise detection of cancer markers. However, the effectiveness of OI is limited by the depth of penetration and the potential issues of dye photobleaching and blinking.

**Ultrasound imaging** employs sound waves to create images and is often used with tagged nanobodies conjugated to contrast agents, such as microbubbles, to visualize systemic vasculature. For instance, anti‐VEGFR2 nanobodies linked to microbubbles have been utilized for imaging angiogenesis in tumors.^138^, ^139^ This non‐invasive technique provides real‐time imaging, allowing for dynamic observation of biological processes. However, ultrasound imaging has lower resolution compared to PET and SPECT, which can limit the detail and clarity of the images.

Additionally, the stability and solubility of nanobodies facilitate their use under harsh conditions, such as high temperatures, extreme pH levels or presence of organic solvents. This nanobody robustness is beneficial for nanobody manufacturing and long‐term storage. It makes them particularly attractive for developing rapid diagnostic tests, employing methods such as lateral flow immunoassays (FLT), diagnostic ELISAs, and biosensors (Figure 3B). In Alzheimer's disease, nanobody‐based rapid testing have been designed specifically testing Aβ and Tau.^140^ They also became popular to detect viral infections caused by norovirus, SARS‐CoV‐2 and MERS^141^, ^142^; or bacterial infections caused by *Salmonella*,^143^ *Escherichia coli* ^144^ or *Bacteroides fragilis* ^145^; or parasitic infection caused by *Trypanosoma congolense* ^146^ or *Toxocara cana*. Additionally, nanobodies facilitate the swift detection of serum C‐reactive protein.^147^ The main finding of the reference is that the ALFA‐tag is a highly versatile epitope tag that, when combined with its specific nanobody (NbALFA), enables a broad range of applications, including super‐resolution microscopy, immunoprecipitations, western blotting, and in vivo detection of proteins, outperforming established tags in versatility and performance.^148^ Besides assisting with early‐stage disease diagnosis, rapid monitoring and monitoring disease progression are also indispensable. To monitor tumor's immune infiltration, nanobodies have been developed to screen for T cell infiltration, and presence of fibronectin.^149^, ^150^

### 3.4 Therapeutic applications of nanobodies in other disease treatment

The applications of nanobodies have not been limited to the treatment of infectious diseases. For instance, in atherosclerosis, anti‐Gal‐2 nanobodies have been shown to reduce plaque size and slow down the progression of plaque buildup.^151^ Additionally, toxins derived from plants and animals have been targeted by nanobodies. For plant toxins, nanobodies are being assessed for neutralizing phytotoxins like ricin and acacia toxin.^115^, ^152^ In the case of animal toxins, nanobodies are being used against snake venom or scorpion toxins.^153^, ^154^ Furthermore, algal toxins such as microcystin and nodularins are also being considered as targets for nanobody‐based treatments.^115^ (Figure 3B).

### 3.5 nanobodies as intracellular probes: Techniques, versatility, and applications in research and medicine

#### 3.5.1 Intracellular targeting methods

Intracellular targeting of nanobodies can be achieved primarily through two methods: transfection of nanobody encoding expression vectors into cells or nanobody protein delivery combined with cell permeability enhancements. Nanobodies can be genetically encoded and fused to genes for fluorescent proteins (FPs) cloned in expression vectors that, after transfection, generate “chromobodies” within cells to target and visualize intracellular target proteins. This procedure enables live‐cell imaging and tracing target‐protein mobility. For instance, chromobodies have been used to reveal the cytoskeleton and nuclear components in real‐time.^155^ Additionally, nanobodies can be attached to cell‐penetrating peptides (cCPPs) to facilitate direct cytoplasmic entry avoiding endocytosis, thereby enhancing labeling efficiencies and enabling their use in drug delivery. An example of this approach includes using cCPP‐modified nanobodies to deliver therapeutic agents directly into cells, improving the targeting and efficacy of treatments.^156^

#### 3.5.2 Therapeutic applications

The autophagy‐targeting nanobody chimeras (ATNCs) have been developed as a versatile tool to selectively degrade intracellular unligandable and undruggable proteins, demonstrating potential therapeutic applications such as suppressing ovarian cancer cell proliferation and migration by targeting the HE4 protein.^157^ The small molecule‐nanobody conjugate inducers of proximity (SNACIPs) have been developed to modulate intracellular processes and endogenous unligandable targets, showcasing potential for therapeutic interventions by controlling cellular functions and inhibiting cancer cell proliferation through targeted mechanisms such as ferroptosis and microtubule nucleation regulation.^158^ A cell‐permeant nanobody‐based degrader targeting BCL11A effectively induces fetal hemoglobin expression, offering a potential therapeutic approach for hemoglobin disorders such as sickle cell disease and β‐thalassemia by enabling the targeted degradation of previously undruggable proteins.^159^

#### 3.5.3 Delivery techniques

The laser‐induced photoporation enables efficient and low‐toxicity delivery of labeled nanobodies into living cells, allowing for high‐quality, long‐term live‐cell microscopy of specific subcellular structures.^160^ Using delivery methods such as electroporation, photoporation, and microfluidic cell squeezing facilitating entry into cells enable Chromobodies or fluorescently labeled nanobodies to visualize dynamic processes of their target within living cells. Additionally, their small size minimizes the distance between the target and the fluorescent label, enhancing resolution in super resolution microscopy (SRM). For instance, Chromobodies have been used to track actin dynamics in living cells, while fluorescently labeled nanobodies have provided high‐resolution images of synaptic proteins using STORM (Stochastic optical reconstruction microscopy).

#### 3.5.4 Imaging applications

nanobodies can be produced in high yields in bacterial systems, purified and then conjugated to chemical dyes, making them suitable for SRM and other advanced imaging techniques. By using dye labeled nanobodies as direct probes for imaging endogenous proteins there will be no need for secondary antibodies. These modified nanobodies also reduce the linkage error and improve the resolution of imaging studies. For example, fluorescently labeled nanobodies have been utilized to visualize microtubule structures with high precision in SRM and have also been employed in tracking the dynamics of proteins involved in cellular signaling pathways. The application of nanotags and nanobodies for live cell single‐molecule imaging enables detailed visualization and analysis of the Z‐ring dynamics in *E.coli*, offering improved resolution and reduced perturbation compared to traditional fluorescent protein fusions.^161^

#### 3.5.5 Advanced biosensors

NanoB2 combining nanobodies with NanoBRET facilitates real‐time quantitative studies of ligand‐membrane protein interactions. Thereby equilibrium and kinetic binding parameters even at endogenous receptor expression levels are determined with high sensitivity.^162^ V5‐tag‐directed nanobody (NbV5) has been developed and optimized to function as an intracellular biosensor, enabling the monitoring of G protein‐coupled receptor (GPCR) signaling and dynamic protein–protein interactions with minimal perturbation to the native cellular environment.^163^ The Intra Q‐body probe, derived from a monoclonal nanobody, effectively targets and visualizes intracellular p53 in living cells with low background noise, enabling precise live‐cell imaging and sorting based on p53 expression levels.^164^

#### 3.5.6 Tools for specific protein studies

A nanobody‐based toolset has been developed to monitor and modify the mitochondrial GTPase Miro1. This engineered nanobody offers new opportunities for studying Miro1's functional roles in mitochondrial dynamics and neurological diseases through advanced biochemical, imaging, and intracellular modulation approaches.^165^ Moreover, a versatile nanobody‐based toolkit has been reported to analyze retrograde transport from the cell surface, allowing for detailed tracking of protein cargo and providing insights into the role of the AP‐1/clathrin machinery in this transport process.^166^

nanobodies serve as powerful tools for intracellular probing enabling targeting of endogenous proteins avoiding extensive modifications. This makes them invaluable for both basic research and clinical applications, providing detailed insights into cellular processes and protein functions.

### 3.6 Clinical success and technological advancements in nanobody‐based treatments

Transitioning from the exciting results observed in laboratory settings, some nanobody‐based products have successfully been translated into rigorous clinical trial processes and received regulatory approval. Cablivi, developed by Ablynx (now part of Sanofi), was the first nanobody‐based drug to receive regulatory approval, obtaining marketing authorization by the European Medicines Agency (EMA) on August 31, 2018^167^ and the U.S. Food and Drug Administration (FDA) on March 15, 2019 (Figure 3C).^168^ It is used for the treatment of acquired thrombotic thrombocytopenic purpura (aTTP) and works by targeting von Willebrand factor (vWF), inhibiting its interaction with platelets, thus preventing the formation of microthrombi.

Another humanized HCAb equipped with a nanobody, Envafolimab (Envida), is the first subcutaneously injectable anti‐PD‐L1 antibody for treatment of microsatellite instability high (MSI‐H) and mismatch repair deficiency (dMMR) advanced solid tumors, approved by China's National Medical Products Administration (NMPA) in November 2021 (Figure 3C).^169^ It also received a fast track designation from the U.S. FDA to treat patients with advanced unresectable sarcomas. Envafolimab works by blocking the PD1‐PD‐L1 interaction and modulating the immune response that leads to an anti‐tumor immune response.^170^, ^171^

Two more nanobody‐based drugs were approved successively in 2022 (Figure 3C), namely Carvykti (Ciltacabtagene Autoleucel) and Ozoralizumab (Nanozora). Carvykti is a chimeric antigen receptor T‐cell (CAR‐T) therapy developed by Janssen‐Cilag International N.V., obtaining marketing authorization by both U.S. FDA and EMA in February and May 2022, respectively, for the treatment of adults with relapsed or refractory multiple myeloma (RRMM) who have received four or more prior lines of therapy.^172^, ^173^ Ozoralizumab, developed by Taisho Pharmaceutical Co. Ltd, is a bispecific nanobody designed to treat rheumatoid arthritis and approved by Japan's Pharmaceuticals and Medical Devices Agency (PMDA). It simultaneously targets tumor necrosis factor‐alpha (TNF‐α) and human serum albumin to extend its half‐life in circulation and to enhance its therapeutic efficacy (Figure 3C).^174^, ^175^

The impressive therapeutic effects of these nanobody drugs have not only advanced the treatment fields of their respective targeted diseases, but they also promoted the recognition and widespread acceptance of nanobody biologic medications in the medical field.

#### 3.6.1 Technological Advancements and Future Prospects

The expansion of the application fields for nanobodies and their accelerated commercialization brings about the acceptance, the upgrading and the change of technology. Enhancing the affinity and stability of nanobodies will improve the application performance. Constructing multivalent therapeutic and diagnostic nanobodies has consequently became a standard procedure. For example, the research on bivalent and trivalent nanobodies targeting the SARS‐COV‐2 has seen significant growth in the years following the outbreak of COVID‐19, yielding ample results with highly mature nanobody constructs.^107^, ^176^ Specifically, bivalent, tetravalent, pentavalent, and even decameric nanobody constructs of higher potency than the monomeric form have been successfully generated.^108^, ^177^, ^178^

To enhance the overall stability of nanobodies, incorporating them into highly stable protein frameworks, such as ferritin scaffolds, has also been assessed.^179^, ^180^ Advances in computational methods and data mining techniques have facilitated the development of synthetic or naïve VHH library constructions as well, allowing researchers to design synthetic libraries based on existing sequence and structural information.^181^ These nanobody libraries are useful in cases where immunization of camelids is not an option for example due to the unavailability of antigen or lack of immunogenicity. The selection methods of target specific nanobodies has also been expanded by using deep sequencing and mass‐spectrometry identifications instead or in combination with phage or yeast display. The modes of nanobody delivery are also enlarged, including intravenous injection, intramuscular injection, nasal inhalation, and oral administration.^108^

The robust growing trends in nanobody technologies are reassuring scientists for its potential to revolutionize medical technology in the future.

## 4 CONCLUSIONS

The evolution of camelid HCAbs in camelids is a testament to nature's ingenuity in adapting immune systems to changing ecosystems. Possibly the requirement of a new antigen‐binding format necessitated camelids to evolve HCAbs to survive a pathogenic treat. The selective advantages of this format must have been enormous to explain the wide spreading and maintenance of these unique homodimeric antibodies across all camelids. However, the unique HCAb structure and its antigen binding by virtue of a minimal‐sized, single antigen‐binding domain have unlocked new possibilities in biotechnology and medicine, leading to the development of nanobodies with translational potential.

nanobodies are revolutionizing therapeutic approaches, offering targeted treatments for cancer and neurological disorders, and infectious diseases. Their small size and stability enhance drug delivery and diagnostic imaging, making them invaluable in early detection and personalize medicine. Nanobody therapies that reached the market, highlighted their impact on patient care, thereby underscoring the transformative potential of nanobodies in modern medicine.

As research advances, collaborations between academic institutions and industry will likely accelerate innovation, providing solutions for more sophisticated applications. With ongoing clinical successes and technological breakthroughs, nanobodies are expected to play a pivotal role in the future in understanding complex biological systems, or in healthcare improving patient outcomes.

## CONFLICT OF INTEREST STATEMENT

The authors declare that the research was conducted in the absence of any commercial or financial relationships that could be construed as a potential conflict of interest.

## Data Availability

These data were derived from the following resources available in the public domain. The structure coordinates used in review are available in Protein Data Bank at https://www.rcsb.org/ and the patents statistics used in the review are available in the LENS.ORG patent database at https://www.lens.org/.
